# The indigestible core of forage fiber: estimation from long-term in vitro kinetics and prediction from lignin content

**DOI:** 10.1093/jas/skag280

**Published:** 2026-08-31

**Authors:** Raphael dos Santos Gomes, Renan Marvila da Silva Santos, Jhone Gleison de Oliveira, Cleber Siqueira Eduardo Júnior, Scizínio Rohem Simão, Munyr Silva Macedo, Luis Orlindo Tedeschi, Ricardo Augusto Mendonça Vieira

**Affiliations:** Campus Colorado do Oeste, Instituto Federal de Educação, Ciência e Tecnologia de Rondônia (IFRO), Colorado do Oeste, Rondônia 76993-000, Brazil; Graduate Program in Animal Science, Universidade Estadual do Norte Fluminense Darcy Ribeiro (UENF), Rio de Janeiro 28013-602, Brazil; Graduate Program in Animal Science, Universidade Estadual do Norte Fluminense Darcy Ribeiro (UENF), Rio de Janeiro 28013-602, Brazil; Graduate Program in Animal Science, Universidade Estadual do Norte Fluminense Darcy Ribeiro (UENF), Rio de Janeiro 28013-602, Brazil; Cargill/Nutron—Departamento de Bovinos de Corte, Cargill Alimentos Ltda, Campinas 13091-060, Brazil; Laboratório de Zootecnia (LZO), Universidade Estadual do Norte Fluminense Darcy Ribeiro (UENF), Rio de Janeiro 28013-602, Brazil; Laboratório de Zootecnia (LZO), Universidade Estadual do Norte Fluminense Darcy Ribeiro (UENF), Rio de Janeiro 28013-602, Brazil; Laboratório de Zootecnia (LZO), Universidade Estadual do Norte Fluminense Darcy Ribeiro (UENF), Rio de Janeiro 28013-602, Brazil; Department of Animal Science, Kleberg Center, Texas A&M University, College Station, TX 77843-2471, United States of America; Laboratório de Zootecnia (LZO), Universidade Estadual do Norte Fluminense Darcy Ribeiro (UENF), Rio de Janeiro 28013-602, Brazil

**Keywords:** digestion kinetics, parametric bootstrap, rumen fermentation

## Abstract

The indigestible core of forage fiber, quantified as indigestible neutral detergent fiber (iNDF, also denoted parametrically as Ω, critically influences ruminant intake, rumen fill, digestibility, and microbial substrate availability. This study estimated digestion kinetics and Ω for 17 forage species (nine grasses and eight legumes) using long-term in vitro incubations and developed a scaling function for Ω prediction from the Lignin (sa): ash-free NDF (aNDFom) ratio. Multivariate analysis of chemical composition (dry matter [DM], crude protein [CP], crude fat, ash, aNDFom, Lignin (sa), and non-fibrous carbohydrates) revealed distinct grass–legume profiles, with grasses showing greater aNDFom (592–763 g/kg DM) and lesser CP (76–166 g/kg DM) than legumes, and lignin ratios ranging 40–230 g/kg aNDFom. Incubations (up to 2,500 h, with medium and inoculum renewal every 96 h) produced biphasic degradation profiles that were fitted with nonlinear mixed models (EXP1, EXP2, GMM1, GMM2, and compartmental GηG1 and GηG1G1) in R (*nlme*). Information-theoretic criteria selected the heterogeneous two-pool compartmental model G6G1G1, which incorporated specimen-specific parameters, random block effects on the lag rate, power-of-the-mean variance, and a continuous autoregressive correlation structure. Fast pools ranged from 140–571 g/kg, slow pools from 59–379 g/kg, with corresponding degradation rates of 0.033–0.128 h⁻¹ and 0.0026–0.0102 h⁻¹. Parametric bootstrap (3,000 replicates) indicated that approximately 300 h of incubation is sufficient to capture 95% of potentially digestible fiber, despite uncertainty in the slow pools. The scaling law Ω = 2.22(Lignin (sa) ⁄ aNDFom)^1.06^ best predicted the indigestible residue, with diagnostics confirming model adequacy. Limitations include the modest number of forage specimens and residual uncertainty in the slow pools. This work integrates kinetic and chemical predictors to improve feed evaluation and ration formulation for ruminants.

## Teaser

This study combined long-term in vitro digestion kinetics with the chemical composition of 17 tropical and temperate forages to develop a simple scaling law that predicts the indigestible fiber core directly from the lignin-to-fiber ratio. The equation, supported by a generalized compartmental model, reveals a resilient isometric relationship and offers a practical alternative to lengthy incubations. These findings provide a faster, more accurate tool for evaluating forage quality and improving ration formulation in ruminant production systems.

## Introduction

Forage plants constitute the foundation of ruminant nutrition worldwide, providing at the same time bulk for normal rumen function and dietary energy through microbial fermentation in the rumen, where complex polysaccharides in the plant cell wall are converted into volatile fatty acids (VFAs) that fuel the metabolism of the host animal (Van Soest 1994; Tedeschi and Fox 2020). The plant cell wall, primarily composed of cellulose, hemicellulose, and lignin, represents the fibrous matrix that occupies space in the gastrointestinal tract (GIT) and, because of its physico-chemical characteristics, forms a raft with multidimensional pools and multiphasic degradation patterns, yet resists digestion by the host’s endogenous enzymes (Van Soest 1994; Mertens 2002; Silva et al. 2018; Tedeschi et al. 2023; Hall and Mertens 2023). However, not all cell wall components are equally accessible or degradable. Lignin is a phenolic polymer that forms cross-links with hemicellulose that interacts directly with cellulose, creating a recalcitrant barrier that limits microbial attachment and enzymatic hydrolysis (Jung et al. 1993; Wilson 1993; Tedeschi et al. 2023; Dhakal et al. 2025). This indigestible “core” of forage fiber (Fisher et al. 1989), often quantified as the indigestible neutral detergent fiber (iNDF; Chandler et al. 1980; Lippke et al. 1986; Robinson et al. 1986; Vieira et al. 2012), directly influences rumen fill, passage rates, intake regulation, and overall digestibility.

Although the mean retention time of fiber in the rumen is typically much shorter than the incubation periods required to approach the true asymptotic residue, a substantial proportion of forage particles remains in the ruminoreticulum for considerably longer periods. Consequently, the size of the asymptotic indigestible fraction continues to influence rumen fill, the effective digestibility of the potentially digestible fiber, microbial protein yield, and voluntary intake. Dynamic models have shown that variation in the indigestible fraction has a nearly proportional effect on maximum digestible dry matter (DM) intake, often greater than the effect of digestion rate itself (Mertens and Ely 1979; Mertens 1987; Mertens and Grant 2020). Accurate estimation of this asymptotic residue is therefore required for realistic predictions of fill, microbial yield, and nutrient supply, even when practical feeding models operate on time scales closer to normal retention times. In applied settings, extended incubations of approximately 240–300 h are commonly used as operational approximations of this asymptotic value (Huhtanen et al. 2006; Krizsan and Huhtanen 2013; Mertens 2016; Palmonari et al. 2016).

The concept of indigestible fiber has evolved since early studies recognized that fiber is not a homogeneous entity but a heterogeneous mixture of fractions with varying nutritional behaviors (Lucas 1964; Van Soest 1967), underscoring the need to separate digestible and indigestible pools for precise evaluation of feeds, forages, and fibrous foods for ruminants (Robinson et al. 1986; Van Soest 1994; Van Soest et al. 2005). Advances in analytical techniques, including ash-corrected neutral detergent fiber (aNDFom; amylase and Na_2_SO_3_-treated, ash-corrected neutral detergent fiber), have improved specificity, but challenges persist in accounting for matrix-specific interactions and heteroscedasticity in measurements (Silva et al. 2018; Hall and Mertens 2023). Mid-term in vitro incubations, typically spanning 96–144 h, simulate ruminal conditions to estimate indigestible residues by exhausting digestible pools, yet they are resource intensive and susceptible to microbial exhaustion or buffer depletion if not correctly managed (Lippke et al. 1986; Robinson et al. 1986; Ellis et al. 2005; Foster et al. 2023).

Lignin’s role as a predictor of indigestibility has been a focal point, with early work establishing empirical ratios such as the Lignin (sa):aNDFom ratio (Chandler et al. 1980; Traxler et al. 1998; Van Soest et al. 2020). Although Vanzant et al. (1998) provided comprehensive recommendations for the standardization of in situ procedures, subsequent evaluations indicate that substantial variation within and among laboratories persists (Foster et al. 2023). Consequently, estimates of the indigestible residue obtained by in situ methods can still be sensitive to procedural details. Recent reviews emphasize the need for forage-specific models, as lignin’s deposition in cell walls reduces the utilization of fibrous carbohydrates, affecting microbial growth and VFA yields (Tedeschi et al. 2023; Dhakal et al. 2025).

Kinetic modeling of fiber digestion has progressed from simple exponential decay to multicompartmental frameworks that address heterogeneity (Smith et al. 1972; Waldo et al. 1972; Mertens 1977; Robinson et al. 1986; Ellis et al. 2005; France et al. 2005; Vieira et al. 2008a). Early models, such as single-pool exponential models, assume homogeneous degradation and do not explicitly account for the lag phases and biphasic patterns observed in degradation profiles (Smith et al. 1972; Waldo et al. 1972; Mertens 1977, 2005). Generalized Michaelis-Menten (GMM) models, first introduced by Groot et al. (1996) into rumen kinetic studies and later presented in equivalent form by France et al. (2005), introduced a shape parameter that greatly improved fit and flexibility for heterogeneous degradation profiles. Compartmental models incorporating gamma-distributed time dependency (GηG1, GηG1G1) better simulate substrate preparation and degradation, ensuring monotonic curves and avoiding nonbiological oscillations (Vieira et al. 2008a, 2012). These models align with Lucas’s heterogeneity by partitioning fiber into fast- and slow-digestible and indigestible pools, enabling predictions of ruminal fiber pools, microbial N flow, and total tract digestibility through the integration of passage kinetics (Vieira et al. 2008b, 2020, 2024). However, limitations include parameter uncertainty in small datasets, methodological inconsistencies in fiber analysis (Silva et al. 2018; Hall and Mertens 2023), and the need for empirical in vivo evaluation, as in vitro methods may overestimate indigestible fractions due to incomplete simulation of the GIT (Huhtanen et al. 2008a; Allen 2019).

Despite these advancements, gaps remain in linking chemical composition to kinetics for diverse forages. Long-term incubations are essential to exhaust slow pools and estimate indigestible residues accurately, but practical alternatives like scaling functions from lignin ratios could streamline predictions for ration formulation and research applications (Van Soest et al. 2005; Mertens 2016; Tedeschi and Fox 2020; Vieira et al. 2024).

This study addresses these gaps by estimating digestion kinetics for 17 tropical and temperate forages using advanced nonlinear mixed models, identifying the best-supported model(s) for heterogeneous profiles, and developing a scaling function to predict indigestible residues from lignin ratios. Specific objectives were 1) to determine chemical compositions and evaluate kinetic parameters via long-term in vitro incubations, 2) to compare model classes (exponential, GMM, compartmental) using information-theoretic criteria, 3) to compute standardized fiber pools and degradation times with uncertainty quantification, and 4) to derive a scaling law for indigestible residue prediction and assess its practical utility in nutrition models. By integrating empirical data with kinetic theory, this work advances forage evaluation to support improvements in ruminant nutrition and feeding.

## Materials and methods

All procedures on animal use and care in this research were approved by the Institutional Committee on Animal Use Ethics (CEUA/UENF) under protocols 380 and 541.

### Characterization of feeds

Seventeen forage specimens from the Poaceae and Fabaceae families were evaluated. Fresh forage samples included Bluestem (*Andropogon gayanus* Kunth cv. Bisquamulatus), Palisade grass (*Urochloa brizantha* (Hochst. ex A. Rich.) R. Webster cv. MG-5), Coastal bermudagrass (*Cynodon dactylon* (L.) Pers.), Elephant grass (*Pennisetum purpureum* Schumach. cv. Cameroon), Guineagrass (*Urochloa maxima* (Jacq.) R. Webster cv. Massai), Swaziland finger grass (*Digitaria swazilandensis* Stent), corn (*Zea mays* L.) silage, corn snaplage silage, and the legumes Pinto peanut (*Arachis pintoi* Krapov. & W.C. Greg. cv. Belmonte), Tropical kudzu (*Pueraria phaseoloides* (Roxb.) Benth.), Perennial horsegram (*Macrotyloma axillare* (E. Mey.) Verdc.), Zarzabacoa galana (*Desmodium adscendens* (Sw.) DC.), Florida hammock milkpea (*Galactia striata* (Jacq.) Urb.), and Bluepea (*Clitoria ternatea* L.).

These forages were harvested at the mid-vegetative stage from experimental plots in Seropédica (22°46′S, 43°41′W, 26 m altitude) and Campos dos Goytacazes (21°43′S, 41°20′W, 16 m altitude), Rio de Janeiro State, Brazil (Aw climate, Köppen classification). Corn was harvested when kernels were approximately one-third milk line (with an approximate DM content of 300 g/kg, as is). Additionally, alfalfa (*Medicago sativa* L.), Tifton 85 bermudagrass (*Cynodon* spp.), and ryegrass (*Lolium perenne* L.) hays harvested at the mid- to late-vegetative stage produced near Dois Vizinhos (25°42′S, 53°05′W, elevation 540 m, Cfa climate, Köppen classification), Paraná State, Brazil, were included. All fresh forages were dried at 55 °C for 72 h in a forced-air oven and, together with the hay samples, ground to pass a 1-mm screen.

### Chemical analysis of forage samples

Forage samples were analyzed for DM (AOAC Method 967.03), crude fat (CF; Thiex et al. 2003), and ash (AOAC Method 942.05). Crude protein (CP) was determined according to the guidelines described in AOAC Methods 984.13 and 2001.11, including N recovery using NH₄H₂PO₄ and lysine-HCl (Thiex et al. 2002; AOAC 2016). Amylase-treated, ash-free neutral detergent fiber (aNDFom) was measured according to AOAC Method 2002.04 using sodium sulfite (Na₂SO₃) and standardized heat-stable amylase solution (Mertens 2002). Lignin (sa) was determined by treating the acid detergent residue with 72% sulfuric acid (Möller 2009). The Lignin (sa):aNDFom ratio (Ratio) was expressed on an aNDFom basis (g/kg). Nonfibrous carbohydrate (NFC) was calculated by difference as NFC = 1,000 − (CP + CF + ash + aNDFom) and expressed on a DM basis (g/kg).

### Long-term in vitro gravimetric incubation

In vitro incubations were conducted in a water bath at 39 °C. Amber 100 mL serum bottles were sealed with methyl rubber stoppers and aluminum crimp seals. Approximately 0.5 g of forage sample (DM basis) was incubated with 40 mL of reduced culture medium and 10 mL of ruminal inoculum. The culture medium, reducing solution, and ruminal inoculum were prepared according to Hall and Mertens (2008).

Ruminal inoculum was obtained from six mature, castrated male Santa Inês sheep (approximately 60 kg) fitted with rumen cannulas. The donor animals were housed in two pens with three animals each, forming two donor groups. For each donor group, inoculum was prepared as a composite sample from the three animals in that pen. Animals were fed once daily with corn silage and soybean meal (mixture composition: 421 g/kg of NDF and 110 g/kg of CP) in amounts sufficient to meet 1.3× maintenance requirements (AFRC 1993; Tedeschi and Fox 2020), with water and mineral supplement offered for ad libitum intake.

Separate samples of liquid and solid ruminal contents were collected from each pen 2 h after feeding (Hall and Mertens 2008), placed in individual pre-warmed thermal bottles (one for solids and one for liquids per pen), and transported to the laboratory. The time between collection and inoculum preparation did not exceed 1 h. Solids were collected within the ruminal mat, where liquids and solids commingle. Ruminal contents from each donor group were blended for 60 s at a 1:2 ratio (solid content: ruminal fluid) to detach adherent bacteria from feed particles, yielding a more representative microbial population that degrades fibrous particles. The mixture was filtered through eight layers of cheesecloth. The resulting ruminal inoculum was added to the pre-reduced culture medium at a 4:1 ratio (medium: inoculum), maintained at 39 °C under constant CO₂ gassing until transfer to the bottles. Bottles were immediately sealed and placed in the water bath. Bottles were gently shaken twice daily (morning and noon) to mix particles and medium. Continuous mechanical agitation is not always practical in flask-based systems because substrate can adhere to the vessel walls above the liquid level (Hall 2015; citing P.J. Van Soest). Manual swirling at regular intervals is the conventional approach used in the classic Goering and Van Soest (1970) method and was therefore adopted for the crimp-sealed serum bottles used in the present study. In addition, the large number of bottles required for the long-term incubations (up to 2,500 h) and the limited capacity of the available shaking water baths made continuous agitation of all vessels unfeasible.

Two water baths were used simultaneously. In each water bath, duplicate samples of every forage were incubated with inoculum from one of the two donor groups (three cannulated sheep per group; six animals total). The combination of water bath and donor group was considered a random, experimental block (Udén et al. 2012).

Incubation times (*τ*) were 0, 1, 3, 6, 9, 12, 24, 36, 72, 96, 120, 144, 216, 288, 432, 864, 1440, 1872, and 2352 h, with additional points up to 2,500 h for selected species to confirm residue stabilization. Incubation times were selected to provide dense sampling during the early phase of digestion (0–36 h, and up to 96 h) in order to characterize the lag and the fast-degrading pool, followed by progressively wider intervals that allowed accurate description of the slow-degrading pool and the asymptotic indigestible residue (iNDF, here represented by Ω). Additional late time points (up to 2,500 h ≈ 104 d) were included for selected forages to confirm that the residue had fully stabilized. This extended sampling strategy follows the long-term anaerobic fermentation approach used by Chandler et al. (1980), who demonstrated that incubations of 70–90 d or longer are often required to exhaust slowly degradable fiber and obtain reliable estimates of the true indigestible fraction. Similar conclusions regarding the necessity of prolonged incubations to characterize the asymptotic residue were reached by Ellis et al. (2005) and by Robinson et al. (1986), who observed that residual NDF continued to decline for more than 30 d in some feeds. The same rationale underpinned the long-term in situ incubations reported by Vieira et al. (2012). Two independent blocks (runs) were performed, with duplicates and two blank bottles (reduced medium + inoculum only) per block. Samples of all forage species were simultaneously incubated per block for the same *τ* to ensure identical conditions.

To maintain maximum microbial fermentative capacity and prevent exhaustion of buffer power, the incubation medium (including blanks) was renewed every 96 h after the first 72 h. Long-term batch cultures are susceptible to progressive declines in microbial activity and buffering capacity (Grant and Mertens 1992; Hall and Mertens 2008). Periodic renewal of medium and inoculum was therefore adopted to sustain an active fibrolytic population and stable pH, thereby allowing the slow-digesting pools to be fully expressed and the asymptotic indigestible residue to be estimated more reliably (Ellis et al. 2005). The 96 h interval was chosen as a practical compromise that limited the number of interventions while still preventing major deterioration of the fermentation environment.

The renewal procedure was performed as follows. Bottles were removed from the water bath, immersed in an ice bath for rapid cooling, and centrifuged at 1,500×*g* for 15 min at 4 °C in a high-capacity centrifuge (model Supra 22K, Hanil Science Industrial Co., Ltd, Gyeyang-gu, Incheon, South Korea; silicone molds used to hold bottles in the rotor). The supernatant was discarded. Fresh reduced medium with inoculum was then added as described for the initial incubation. Time outside the water bath without medium was subtracted from the total incubation time.

At the end of each τ, bottles were immersed in an ice bath, frozen until analysis, thawed, and centrifuged at 1,500×*g* for 15 min. The supernatant was filtered through Whatman No. 41 ashless quantitative filter paper (fast filtration speed, pore size approximately 20–25 µm) with minimal particle transfer. Particles adhering to the paper and the remaining particles in the serum bottle were quantitatively transferred with 50 mL of neutral detergent (additional detergent used when needed) into 600 mL Berzelius beakers without spouts and boiled for 1 h. The contents were transferred back to serum bottles, centrifuged repeatedly with hot water until detergent free (approximately five cycles), transferred to crucibles with acetone, dried by suction, oven-dried overnight at 105 °C, weighed, incinerated, and finally weighed at room temperature. Residues at mid-incubation times or longer became difficult to filter; therefore, 15 g of muffle-burned sand was added as a filter aid (Mertens 2002). For fibrous residues from 864 to 2,352 h, contents were obtained by centrifugation (1,500×*g* for 15 min at room temperature) after the 1 h boiling step, with the boiled material transferred directly into serum bottles. At the end of each cycle, boiling water was used to transfer loosened particles from the filter paper back to the serum bottle; their weight was balanced with hot distilled water, and the cycle was repeated five times. The final residue was transferred to a Gooch crucible, treated with acetone, dried by suction, oven-dried overnight at 105 °C, weighed, and muffled for ash correction.

For comparison with published long-term incubation data (Chandler et al. 1980; Vieira et al. 2012), ash-corrected residual NDF at each time point (including time zero) was obtained without Na₂SO₃ (Van Soest et al. 1991). Residues were blank-corrected and expressed as g/kg of NDF at time zero (blanks were also ash-corrected). The general term “NDF” (ash-corrected residual neutral detergent fiber determined without Na₂SO₃) was used for the incubation residues to differentiate them from aNDFom (the standardized amylase- and Na₂SO₃-treated, ash-corrected fiber). The experiment provided two experimental units (blocks), with two replicates for each forage–time combination within block.

### Model descriptions and fitting strategy

We first conjectured that all degradation profiles would exhibit logical long-term trends, with fast and slow pools converging to an asymptote. To test this assumption rigorously, we visually inspected all individual profiles. A total of 260 observations (20.1% of 1,292) were discarded because they exhibited a clear non-biological increase in residual mass, a methodological artifact that occurred primarily at incubation times greater than 500 h (179 of the 260 excluded points). Such anomalous increases violated the expected monotonic decline of residual NDF and were therefore removed. All remaining 1,032 data points retained the biologically expected monotonic pattern and were used for subsequent model fitting.

Long-term NDF degradation profiles were interpreted using two basic model classes: single-pool (homogeneous) models and two-pool (heterogeneous) models, each including an indigestible residue at the asymptotic phase (France et al. 2005; Mertens 2005; Vieira et al. 2008a). The exponential single- and two-pool models were the simplest and were fitted with increasing levels of complexity:


(1) $$R(\tau )=\sum_{i=1}^{I}A_{i}\exp\left(-\kappa_{i}\tau \right)+\Omega .$$

The homogeneous degrading pool (Greek upper case *A* > 0, for *I* = 1, EXP1) degrades at a fractional rate *κ* (expressed as h^−1^). The two-pool version (*I* = 2, EXP2) consists of a fast (*A*_1_) and a slow (*A*_2_) degrading fraction ($\forall A_{i}>0$, expressed as g/kg NDF) that degrade at fractional degradation rates *κ_i_* h^−1^, assuming *κ*_1_ > *κ*_2_ > 0. The asymptotic indigestible residue is denoted by the Greek upper-case letter Ω (g/kg NDF). This parametric notation is used solely for consistency with the other fixed population parameters of the models (*A_i_*, *κ_i_*, *λ*, *η*, *θ_i_* etc.) and does not replace the well-established term iNDF (Huhtanen et al. 2008a; Mertens 2016; Palmonari et al. 2016).

The generalized Michaelis–Menten model (GMM; Groot et al. 1996; France et al. 2005), a flexible solution used to interpret gas production profiles, was rearranged to describe fiber degradation, as follows:


(2) $$R(\tau )=\sum_{i=1}^{I}A_{i}(1-\tau^{\theta_{i}}/\left(\tau^{\theta_{i}}+{Κ}_{i}^{\theta_{i}}\right))+\Omega$$

Analogously, the single pool version (Greek upper cases *A*, *K*, and Ω, and *θ* all > 0) is obtained with *I* = 1 (GMM1), and the two-pool version with *I* = 2 (GMM2). The fast (*A*_1_) and slow (*A*_2_) degrading fractions are half-degraded at *K_i_* (h), *θ_i_* are shape parameters, and Ω represents the indigestible fraction.

The generalized compartmental model of fiber degradation aggregates Gamma time dependency (Matis et al. 1989) into substrate preparation and degradation steps as sequential dynamic events (Vieira et al. 2008a, 2012):


(3) $$R(\tau )=\sum_{i=1}^{I}A_{i}(\delta_{i}^{\eta }\exp\left(-\kappa_{i}\tau \right)+\exp\left(-\lambda \tau \right)\sum_{j=0}^{\eta -1}(1-\delta_{i}^{\eta -j}){\left(\lambda \tau \right)}^{j}/j!)+\Omega$$

with $A_{i}$, $\kappa_{i}$, and Ω (all > 0) having the same definitions described for the exponential model, λ is the fractional rate of substrate preparation to be degraded ($h^{-1}$), and the auxiliary quantity $\delta_{i}=\lambda /\left(\lambda -\kappa_{i}\right)$. η is the dimensionless order of time-dependency $\left(\eta \in Z^{+}\right)$. Important assumptions are $\lambda >\kappa_{i}>0$ and $\eta /\lambda <1/\kappa_{i}$ to ensure monotonic degradation and avoid non-physical oscillations, consistent with the biological expectation that substrate preparation precedes and limits degradation (Mertens 2005; Vieira et al. 2008a, 2012). The single pool version is GηG1 (I=1), and the two-pool (heterogeneous) version (I=2) is described as GηG1G1 (Ellis et al. 2005).

In these models, the Gη term is not a biological pool of substrate. It is a mathematical abstraction that generates an age-dependent rate process describing the progressive preparation of the forage particle for microbial hydrolysis (Matis et al. 1989; Ellis et al. 2005; Vieira et al. 2008a). Once prepared, the substrate enters one or more first-order degradation pools (G1). Consequently, $G_{\eta }G1$ describes a single-pool (homogeneous) model and $G_{\eta }G1G1$ describes a two-pool (heterogeneous) model. The integer η controls only the shape of the preparation-time distribution and does not represent an additional substrate pool.

### Statistical procedures and model fitting

The chemical composition data were analyzed by multivariate analysis of variance (MANOVA) according to the general linear model Y=XB+E, where Y is the n×p matrix containing n–observations by p–variables (analytes), X is the n×q design or incidence matrix, B is q×p matrix of parameters (q is number of parameters, including intercepts and specimen effects), and E is the n×p error matrix. The model assumes $E\;∼\;N_{p}(0,\;\Sigma )$, in which 0 is a p×1 zero vector, and Σ is the positive definite p×p variance-covariance matrix of the errors. The model was fitted using PROC GLM in SAS Studio 9.4 (SAS Institute Inc., Cary, NC, USA) to obtain least-squares means (LSM) and standard errors of the means (SEM) for each analyte and forage species.

We iteratively created r-th model versions from Eq. (1)–(3). First, the single-pool EXP1, GMM1, and GηG1) and two-pool (EXP2, GMM2, and GηG1G1) models were fitted with PROC NLIN (Method = Marquardt) of SAS to obtain initial parameter estimates for each forage species. These estimates were used as initial guesses in the *gnls* function of R. A general version for each model with no specimen-specific effects was fitted first. The family (FABACEAE and POACEAE) effect was then added, followed by specimen-specific forage effects attributed to the model parameters. When necessary, PROC NLMIXED in SAS was used to obtain refined initial estimates for each forage species using the Gauss–Newton method with the Newton–Raphson optimization. These improved guesses better conditioned the models for maximum-likelihood fitting with *gnls* in R.

Variance was modeled under the assumption of normality, independence, and homoscedasticity. However, we also tested the power-of-the-mean variance function (POM, $\sigma_{\Omega }^{2}=\sigma^{2}{\left|R(\tau )\right|}^{2\psi }$) and a continuous autoregressive correlation structure (CAR, parameter ϕ) to address potential heteroscedasticity. Random effects of blocks were attributed to the model parameters via *nlme* function in R. Each model version had a number of parameters $n_{\Theta }$ (including variance and covariance parameters; Pinheiro and Bates 2000; Pinheiro et al. 2025). Model fit was considered valid if the algorithm converged, yielded a positive-definite variance-covariance matrix, produced no heavy-tailed confidence intervals for random effects, and satisfied model assumptions (e.g., $\hat{\lambda }>\hat{\kappa }$).

The Information-Theoretic (I-T) approach provided the framework for multimodel inference (Burnham and Anderson 2004). The Akaike information criterion (Akaike 1974) corrected for small samples (${\mathrm{AICc}}_{r}$; Hurvich and Tsai 1989; Cavanaugh 1997), Akaike differences $\left(\Delta {\mathrm{AICc}}_{r}\right)$, model probabilities (or Akaike weights, $w_{r}$), and evidence ratios $\left({ER}_{r}\right)$ were computed for each successfully fitted r-th model version. When model uncertainty $\left(1-w_{r}\right)$ was substantial ($\max\;w_{r}<0.90$), model averaging was considered; otherwise, inference was based on the single best-supported model $\left(\max\;w_{r}\geq 0.90\right)$.

Given the best-supported model solution(s) from the I-T approach, additional parameters were estimated using a two-step procedure for inferential purposes (Oliveira et al. 2020; Vieira et al. 2020). Standardized fractions were computed and reported (e.g., $A^{\mathrm{std}}=A/\left(A+\Omega \right)$, $A_{2}^{\mathrm{std}}=A_{2}/\left(A_{1}+A_{2}+\Omega \right)$). Parameter estimates obtained from *gnls* or *nlme* in R served as inputs to SAS NLMIXED, allowing estimation of additional parameters via the Gauss–Hermite quadrature method without optimization (TECHNIQUE = NONE).

One challenge in empirically estimating the indigestible residue is defining the incubation time required for near-complete exhaustion of potentially degradable substrates (Lippke et al. 1986; Robinson et al. 1986; Lund et al. 2004; Casali et al. 2008). Single-pool versions of Eqs. (1) and (2) allow explicit (analytical) solutions for τ. However, the single-pool $G_{\eta }G1$ and the dual-pool versions of Eqs. (1)–(3) are transcendental and lack analytical solutions. Therefore, parameter estimates and their standard errors from the best-supported model(s) were used to determine numerically the times (τ) at which 95% and 99% of the potentially degradable fiber is exhausted.

Medians ${\tilde{\tau }}_{95\%}(\Omega )$ and ${\tilde{\tau }}_{99\%}(\Omega )$ and 95% confidence intervals (95%BCa) were obtained using a Monte Carlo parametric bootstrap, customized with the *replicate* and *rnorm* functions in R (fixed effects and their standard errors from *nlme*). The *uniroot* function in R was used to find the $\tau_{95\%}(\Omega )$ and $\tau_{99\%}(\Omega )$ roots of f(τ)=R(τ)-TR (where TR is the target residue) with search bounds from 0 to 30,000 h. Roots are τ values satisfying $R(\tau )=\Omega +0.05(A_{1}+A_{2})$ as the TR for $\tau_{95\%}(\Omega )$, and $R(\tau )=\Omega +0.01(A_{1}+A_{2})$ as TR for $\tau_{99\%}(\Omega )$. Medians and the 95%BCa for the means were generated after 3,000 bootstrap replicates. Possible adverse outcomes for positive-constrained parameters were truncated at zero, and invalid replicates (e.g., $\lambda \leq \max\left(\kappa_{1},\kappa_{2}\right)$) were discarded. The 2.5th and 97.5th percentiles of the resulting bootstrap distributions provided the lower and upper bounds of the 95%BCa. This parametric bootstrap accounted for uncertainty in all fixed effects and variance components, providing robust inferences suitable for small-sample inference in nonlinear mixed models (Pinheiro and Bates 2000).

### Long-term indigestible residue prediction

Researchers have long sought to establish relationships between the indigestible fibrous residue and lignin content in forages (Smith et al. 1972). Chandler et al. (1980) averaged the quotient $\Omega_{\text{Chandler}}/\text{Ratio}$ (where Ratio=lignin/NDF) and obtained an average value of 2.4±0.4. $\Omega_{\text{Chandler}}$ is the refractory NDF fraction after long-term (90–120 d) in vitro incubations in anaerobic fermenters; they determined lignin as the 72% sulfuric acid residue, and NDF without Na_2_SO_3_. In practice, the Chandler’s equation is a scaling function: $\Omega_{\text{Chandler}}=\gamma_{0}{\left(\text{Ratio}\right)}^{\gamma_{1}}$, with ${\hat{\gamma }}_{0}=2.4$, assuming $\gamma_{1}=1$.

Traxler et al. (1998) revised the estimation of indigestible fiber fraction using the relationship between indigestible residue after 96 and 144 h of in vitro digestion (Daisy^II^ System). They modeled it with a power law (scaling) function fitted via log-log regression, which in the arithmetic scale corresponds to $\Omega_{\text{Traxler}}=\gamma_{0}{\left(\text{Ratio}\right)}^{\gamma_{1}}$ g/kg NDF, where ${\hat{\gamma }}_{0}=6.17$ (no SE reported), ${\hat{\gamma }}_{1}=0.77\pm 0.026$, and $\hat{\sigma }=7.22\;$g/kg. Traxler’s lignin was sulfuric acid lignin, and NDF was measured exclusive of residual ash (possibly with heat-stable amylase), but without mention of Na_2_SO_3_ use.

In the present study, a general scaling function was fitted after expanding the vector of observations (study 1) to include those of Vieira et al. (2012), who obtained 70 d residues of forage NDF via in situ incubations (study 2), and Chandler’s data (study 3), for a total of 43 estimates (observations). The scaling function has the general form


(4) $$\Omega =\gamma_{0}{\left(\text{Ratio}\right)}^{\gamma_{1}},$$

where $\gamma_{0}\;(g^{1-\gamma_{1}}/{\text{kg}}^{1-\gamma_{1}})$ is the standard indigestible residue and $\gamma_{1}$ is the dimensionless power. Therefore, Eq. (4) was fitted under seven different conditions: 1) on the arithmetic scale assuming normality and homoscedasticity, with the likelihood integrated via the Gauss-Hermite quadrature and maximized using the Newton–Raphson algorithm with ridging in SAS NLMIXED; 2) assuming normality and the power-of-the-mean variance function (POM), $\sigma_{\Omega }^{2}=\sigma^{2}|\gamma_{0}(\text{Ratio}{)}^{\gamma_{1}}{|}^{2\psi }$, expressed as g²/kg², with σ as $g^{1-\psi }/{\text{kg}}^{1-\psi }$ and dimensionless ψ, under the same optimization; 3) using the Laplace approximation to the likelihood in SAS GLIMMIX assuming a Gamma distribution and a logarithmic link function as the linear predictor: $\log(\Omega )=\log(\gamma_{0})+\gamma_{1}l\mathrm{og}(\text{Ratio})$; 4) using Laplace approximation in GLIMMIX assuming a lognormal distribution, where $\mu =\log(\gamma_{0})+\gamma_{1}l\mathrm{og}(\text{Ratio})$ and $\sigma^{2}$ are in log scale (in the arithmetic scale, $\Omega =\exp(\mu +\sigma^{2}/2)$ and $\sigma_{\Omega }^{2}=\exp(2\mu +2\sigma^{2})-\exp(2\mu +\sigma^{2})$; Vieira et al. 2018); 5) using Laplace approximation in NLMIXED to fit Ω under a lognormal distribution but with a POM variance function in the log scale, i.e., μ as in condition (4) but $\sigma_{\log}^{}=\sigma^{2}|\mu {|}^{2\psi }$ (Vieira et al. 2018); 6) using NLMIXED with Chandler’s reported values as inputs to predict Ω via μ=log⁡(2.428)+log⁡(Ratio) and $\hat{\sigma }=0.4147$ (both estimates obtained via NLMIXED with study 3 data) under a lognormal distribution and no optimization; and 7) using Traxler’s estimated parameters to predict Ω as in condition (6), but with μ=log⁡(6.17)+0.77log⁡(Ratio) and $\hat{\sigma }=\log(7.22)$ as a simple linear regression in the log scale (no assumption regarding lognormal distribution). All seven models were evaluated according to the I-T approach described earlier. An agreement plot was generated using the *AgreementInterval* package of R (Liao 2015) for the best-supported solution(s).

We also used the GLIMMIX procedure to fit Ω under Gamma distribution and log link function to check for possible differences among studies arising from different incubation methods and lignin/NDF analytical procedures. The log link function was as follows:


(5) $$\log\left(\Omega \right)=\xi +\xi_{k}+\gamma_{1}\log\left({\text{Ratio}}_{k}\right)+\gamma_{1k}\log\left({\text{Ratio}}_{k}\right),$$

where ξ is the intercept, $\xi_{k}$ is the k-th study effect, $\gamma_{1}$ is the common slope, $\gamma_{1k}$ is the slope by study, and $\log\left({\text{Ratio}}_{k}\right)$ is the ratio for each observed value of the k-th study.

## Results

The chemical composition of the 17 forage samples studied showed considerable variation across analytes and specimens (Table 1). Least-squares means obtained from MANOVA were justified by the presence of significant (*P* < 0.05) correlations (correlation matrix not shown) among components measured under the same analytical conditions. For example, Lignin (sa) and CP presented a positive, significant (*P* = 0.042) correlation ($\hat{\rho }=0.4835$). The MANOVA test criteria were all significant (*P* < 0.001): Wilks’ lambda, Pillai’s Trace, Hotteling-Lawley Trace, and Roy’s Greatest Root, which means that at least one (likely several) dependent variables differ across forage species, and/or the pattern of correlations among variables (analytes) differs by species. Relative standard deviations (%RSD) ranged as follows: 0.08–0.41% (DM), 0.26–0.82% (CP), 1.89–7.41% (CF), 0.59–2.79% (Ash), 0.08–0.24% (aNDFom), 0.50–5.16% (Lignin (sa)), 0.10–3.20% (NFC), and 0.27–1.41% (Ratio). Components with lesser absolute values generally exhibited greater %RSD, as expected from analytical precision limits. Standard errors of the means (SEM) reflect the multivariate structure (Table 1).

**Table 1:** Least squares means from multivariate analysis on the chemical composition of the studied forages^1^.

Species	DM, g/kg as is	CP, g/kg DM	CF, g/kg DM	Ash, g/kg DM	aNDFom, g/kg DM	Lignin (sa), g/kg DM	NFC, g/kg DM	Ratio
**Bluestem**	201.3	109.7	22.4	77.3	687.8	40.0	103.0	58.2
**Palisade grass**	191.2	134.1	26.9	84.9	592.5	28.8	161.8	48.7
**Coastal Bermudagrass**	311.7	89.8	18.3	70.2	751.8	61.6	70.0	81.9
**Elephant-grass**	157.6	136.4	17.8	97.1	672.9	36.9	75.9	54.9
**Guinea grass (cv. Massai)**	255.3	106.8	23.1	98.2	707.8	31.7	64.3	44.7
**Swaziland finger grass**	203.2	133.0	29.5	84.4	667.2	39.1	86.0	58.6
**Corn silage**	250.7	82.2	27.6	53.0	474.3	31.8	363.1	67.0
**Tifton 85 hay**	808.7	166.2	10.7	61.1	742.5	50.1	19.7	67.3
**Corn snaplage silage**	380.8	76.5	33.4	22.6	260.1	12.2	607.5	46.9
**Ryegrass hay**	825.1	102.0	12.8	66.4	762.8	57.5	56.1	75.3
**Alfalfa hay**	824.3	244.9	15.6	103.1	439.0	76.2	197.5	173.5
**Pinto peanut**	188.6	235.3	8.5	93.7	505.1	101.6	157.6	201.2
**Zarzabacoa galana**	254.5	113.2	15.7	59.6	541.4	125.7	270.1	232.2
**Florida hammock milkpea**	258.1	240.3	12.5	104.0	421.8	75.7	221.5	179.5
**Tropical kudzu**	159.5	214.0	10.6	107.0	516.2	76.1	152.3	147.4
**Perennial horsegram**	152.3	166.5	21.0	82.6	500.7	67.9	229.3	135.5
**Bluepea**	207.4	220.2	31.0	68.6	459.9	76.5	220.5	166.3
**SEM**	0.63	3.8	0.59	0.83	6.7	1.6	8.5	3.0

1 aNDFom = neutral detergent fiber measured with heat-stable amylase, sodium sulfite, and expressed exclusive of residual ash; Lignin (sa) = acid detergent residue treated with 72% sulfuric acid and expressed exclusive of residual ash. Ratio = Lignin (sa):aNDFom ratio on a fiber basis (g/kg).

### Nonlinear models of fiber degradation

Several candidate models were fitted to the degradation profiles, including single- (EXP1) and dual-pool (EXP2) exponential, generalized Michaelis–Menten (GMM1), and various versions of the generalized compartmental models (GηG1 and GηG1G1). The dual-pool, heterogeneous GMM2 model did not fit successfully to the data (errors in *nls* steps or failed convergence) using *gnls* (fixed effects only) and *nlme* (with random effects ascribed to one or more parameters of GMM2). The best exponential model (dual-pool, specimen-specific parameters, homoscedastic variance) had ${\Delta \mathrm{AICc}}_{r}=1019.8$ and negligible support ($w_{r}\approx 10^{-222}$). Similarly, the best homogeneous, single pool version of GMM (specimens-specific parameters, with a POM variance and CAR structure) with no random effects ascribed had ${\Delta \mathrm{AICc}}_{r}=255.9$ and $w_{r}\approx 10^{-56}$. Other GηG1 variants (e.g., G7G1 with random effects on λ and κ, ${\Delta \mathrm{AICc}}_{r}\approx$ 508.9–646.4 and $w_{r}\approx 10^{-111}$) also received no meaningful evidence support, as revealed by I-T criteria, given the data. The top model was unequivocally the biphasic, dual-pool G6G1G1 with specimens-specific parameters, random block effects $\left({\hat{\sigma }}_{\lambda }\right)$ on the dynamic lag parameter (λ), POM variance $\left(\hat{\psi },\;\text{dimensionless}\right)$, CAR structure $\left(\hat{\phi },\;\text{dimensionless}\right)$, and within-class residual standard deviation $\left(\hat{\sigma },\;\text{expressed}\;\text{as}\;g^{1-\hat{\psi }}/{\text{kg}}^{\hat{\psi }-1}\right)$, as demonstrated by the I-T approach: ${\Delta \mathrm{AICc}}_{r}=0$ and $w_{r}\approx 0.9998$, with $n_{\Theta }=106$. This model provided the best description of dynamic lag, biphasic digestion, and asymptotic long-term residue behavior, given the data (Table 2).

**Table 2:** Parameter estimates^1^,^2^ (mean ± SE) of in vitro gravimetric digestion kinetics of neutral detergent fiber (NDF) of 17 forage specimens using the biphasic generalized compartmental model GηG1G1 with η=6.

Species	${Α}_{1}$ **, g/kg NDF**	λ , $\times 10^{-2}$**/h**	$\kappa_{1}$ , $\times 10^{-2}$**/h**	${Α}_{2}$ **, g/kg NDF**	$\kappa_{2}$ , $\times 10^{-2}$**/h**	Ω **, g/kg NDF**
**Bluestem**	530 ± 37	77 ± 8	7.2 ± 1.1	267 ± 35	0.69 ± 0.14	189 ± 12
**Palisade grass**	523 ± 45	76 ± 8	8.5 ± 1.6	272 ± 43	0.94 ± 0.19	160 ± 8
**Coastal Bermudagrass**	428 ± 33	72 ± 10	6.7 ± 1.2	174 ± 32	0.55 ± 0.17	376 ± 9
**Elephant-grass**	571 ± 48	97 ± 12	4.8 ± 0.6	212 ± 46	0.67 ± 0.19	179 ± 11
**Guinea grass (cv. Massai)**	565 ± 57	76 ± 8	7.4 ± 1.3	237 ± 54	1.02 ± 0.27	176 ± 8
**Swaziland finger grass**	532 ± 44	74 ± 8	9.7 ± 1.9	237 ± 42	1.00 ± 0.23	183 ± 8
**Corn silage**	388 ± 45	42 ± 7	5.6 ± 1.5	379 ± 43	0.68 ± 0.12	200 ± 16
**Tifton—85**	525 ± 43	71 ± 10	4.1 ± 0.6	208 ± 42	0.49 ± 0.16	231 ± 16
**Corn snaplage silage**	413 ± 105	39 ± 6	4.0 ± 1.3	366 ± 102	0.87 ± 0.23	197 ± 9
**Ryegrass hay**	508 ± 42	61 ± 9	4.7 ± 0.7	173 ± 40	0.56 ± 0.19	271 ± 8
**Alfalfa**	440 ± 24	64 ± 9	6.9 ± 1.1	97 ± 22	0.39 ± 0.2	412 ± 12
**Pinto peanut**	498 ± 21	71 ± 10	5.2 ± 0.6	95 ± 23	0.23 ± 0.17	414 ± 22
**Zarzabacoa galana**	228 ± 59	62 ± 16	5.7 ± 2.3	164 ± 56	0.84 ± 0.36	605 ± 10
**Florida hammock milkpea**	542 ± 33	1270 ± 2270	3.3 ± 0.3	59 ± 27	0.26 ± 0.33	476 ± 14
**Tropical kudzu**	373 ± 59	89 ± 15	5.7 ± 1.3	149 ± 53	0.83 ± 0.43	457 ± 14
**Perennial horsegram**	438 ± 22	85 ± 11	7.7 ± 1.1	165 ± 27	0.33 ± 0.13	383 ± 24
**Bluepea**	140 ± 27	87 ± 30	12.8 ± 10.1	335 ± 25	0.83 ± 0.14	516 ± 15

1 $A_{1}$ , $A_{2}$ = fast and slow potentially digestible fractions; λ= time-dependent rate related to dynamic lag events; $\kappa_{1}$, $\kappa_{2}$ = respective $A_{1}$ and $A_{2}$ degradation rates; Ω = long-term indigestible fraction.

2 Variance components: ${\hat{\sigma }}_{\lambda }=5.21\times 10^{-2}/h$, ϕ=0.1445 (dimensionless), $\hat{\psi }=0.019$ (dimensionless), and $\hat{\sigma }=22.24g^{1-\hat{\psi }}/{\text{kg}}^{\hat{\psi }-1}$ of NDF.

Representative long-term in vitro degradation profiles of NDF for six contrasting forage species are shown (Figure 1). These profiles illustrate the typical biphasic pattern captured by the G6G1G1 model: an initial dynamic lag phase (highlighted in the insets for the first 50 h), followed by rapid depletion of the fast-digesting pool, slower degradation of the slow pool, and convergence toward an asymptotic indigestible residue. The close agreement between observed residues and model predictions, as well as the small range of confidence intervals, confirms the adequacy of the G6G1G1 structure in describing these heterogeneous digestion kinetics across grasses and legumes.

**Figure 1:**
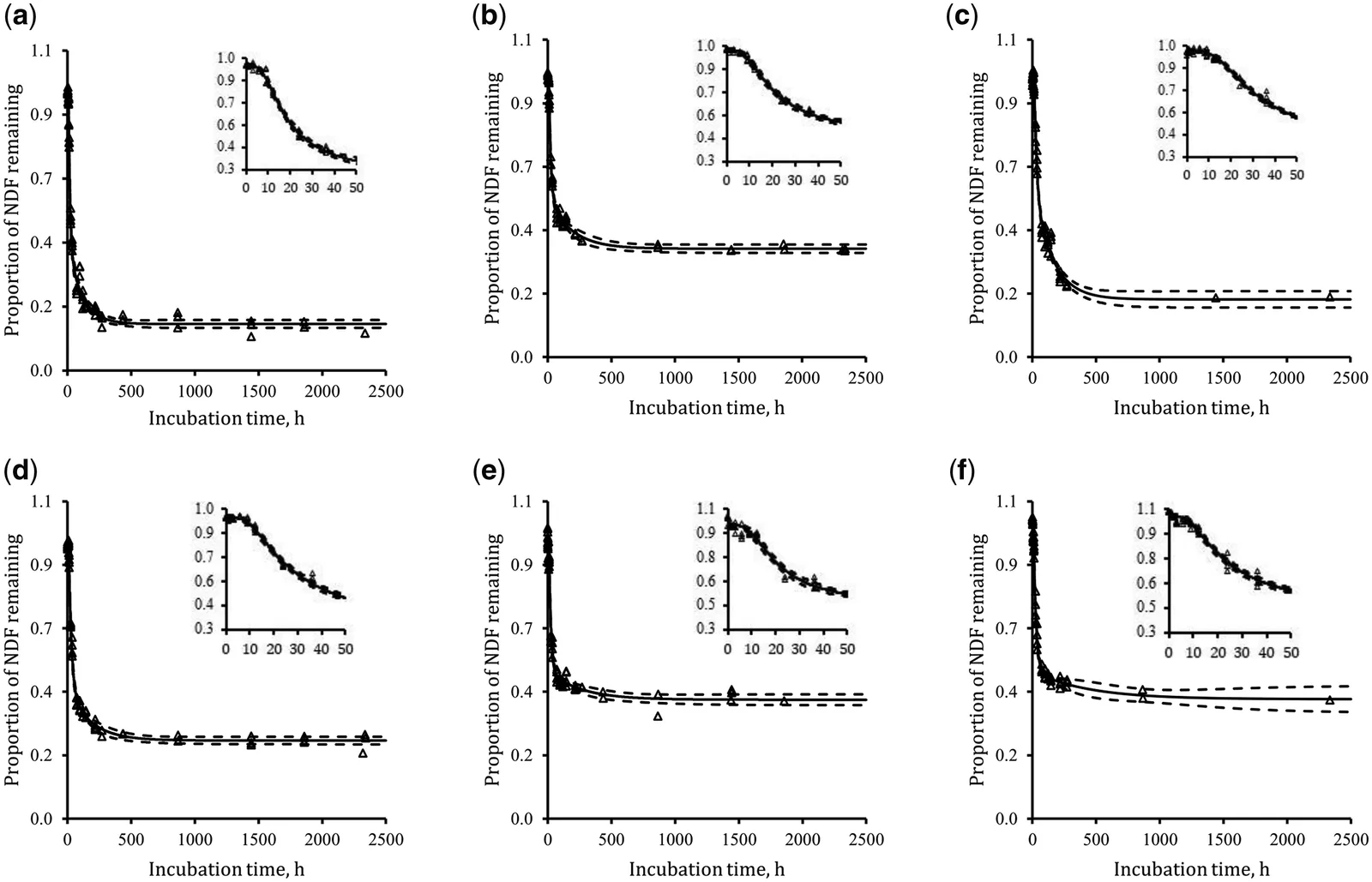
Examples of long-term in vitro degradation profiles of neutral detergent fiber (NDF) for six forage specimens. Panels show observed data (symbols) and fitted lines from the biphasic generalized compartmental model $G_{\eta }G1G1$(η=6). Insets (top right of each panel) highlight the dynamic lag phase during the first 50 h of incubation. a) Palisade grass, b) Coastal Bermudagrass, c) Corn silage, d) Ryegrass hay, e) Alfalfa hay, and f) Pinto peanut.

The fitted G6G1G1 revealed distinct digestion kinetics among the 17 forage species, as revealed by specimen-specific parameter estimates (Table 2). The fast, potentially digestible fraction $\left(A_{1}\right)$ ranged widely, with legumes such as alfalfa and pinto peanut showing lesser values compared to most grasses, whereas the slow fraction $\left(A_{2}\right)$ dominated in elephant-grass, guinea grass, swazi, and corn silage. Digestion rates for the fast-digesting pool $\left(\kappa_{1}\right)$ were generally greater in legumes and some tropical grasses, indicating more rapid initial digestion, whereas the rates of slow-digesting pools $\left(\kappa_{2}\right)$ exhibited greater variability, with lesser values in Tifton, alfalfa, pinto peanut, and horsegram, suggesting prolonged degradation. The mean duration of dynamic lags (η/λ, h) for corn silage and corn snaplage, silage fibers endured for almost 15 h. Lags were intermediary for alfalfa hay, zarzabacoa, and ryegrass hay (∼ 9.6 h), whereas other forage species had their fibers prone to being digested at faster times (∼ 7.5 h). Florida hammock pea presented a large variation on the preparation rate parameter (λ), which yielded a fast mean preparation time for its fibrous substrate (∼ half an hour). Curiously, despite the fast substrate preparation, mean digestion times $\left(\eta /\lambda +1/\kappa_{1}+1/\kappa_{2},\;h\right)$ for hammock milkpea and pinto peanut, surpassed 400 h. Mean digestion times were intermediary (∼ 200–300 h) for bermudagrass, Tifton, alfalfa and ryegrass hays, and horsegram. Fast-digesting pools were practically exhausted in less than 15 h $\left(1/\kappa_{1}\right)$ for bluestem, palisade grass, bermudagrass, guinea grass, and swazi grass. Other forage fiber fast pools took longer mean digestion times (from 17 to 30 h). Slow-digesting pools, on average, took 100 to 200 h, and were particularly longer for legumes, pinto peanut, hammock milkpea, and horsegram.

To avoid bias from experimental conditions, fast- and slow-digesting pools and the indigestible pool were standardized (summing up to one) as new parameter estimates obtained from the NLMIXED procedure (Tables 2 and 3). The standardized fast-digesting pool was generally larger in grasses than in legumes, with bluestem, palisade grass, elephant-grass, swazi, Tifton, guinea grass, and ryegrass exhibiting the highest proportions, whereas legumes such as alfalfa, pinto peanut, and Florida hammock milkpea showed lesser slow pools and greater indigestible residues. The fast- $\left(A_{1}^{\mathrm{std}}\right)$ and slow-digesting $\left(A_{2}^{\mathrm{std}}\right)$ standardized pools in corn silage and corn snaplage silage were relatively balanced (Table 3). The two-step errors estimated for these pools were relatively lesser for most species, reflecting robust estimation, but wider in legumes with high indigestible fractions. However, the %RSD range for the standardized indigestible residue $\left(\Omega^{\mathrm{std}}\right)$ varied from 1.5% to 7.2%, whereas standardized pools $A_{1}^{\mathrm{std}}$ and $A_{2}^{\mathrm{std}}$ had %RSD varying from 2.98% to 18.83%, and from 5.61% to 40.89%, indicating greater parameter uncertainty.

**Table 3:** Standardized parameters^1^ of fast ($A_{1}^{\mathrm{std}}$) and slow ($A_{2}^{\mathrm{std}}$) potentially digestible NDF fractions and long-term indigestible residue ($\Omega^{\mathrm{std}}$), bootstrap median times to reach 95% and 99% of NDF degradation^2^.

Species	$A_{1}^{\mathrm{std}}$ **, g/kg**	$A_{2}^{\mathrm{std}}$ **, g/kg**	$\Omega^{\mathrm{std}}$ **, g/kg**	${\tilde{\tau }}_{95\%}(\Omega )$ **, h (95% BCa)**	${\tilde{\tau }}_{99\%}(\Omega )$ **, h (95% BCa)**
**Bluestem**	538 ± 40	270 ± 38	192 ± 11	283 (199, 483)	517 (366, 880)
**Palisade grass**	548 ± 59	284 ± 55	167 ± 7	213 (149, 356)	385 (274, 644)
**Coastal Bermudagrass**	438 ± 31	178 ± 30	385 ± 8	323 (199, 835)	610 (387, 1553)
**Elephant-grass**	593 ± 43	220 ± 41	186 ± 10	257 (155, 559)	500 (313, 1073)
**Guinea grass (cv. Massai)**	577 ± 91	243 ± 86	180 ± 8	181 (113, 370)	338 (220, 692)
**Swaziland finger grass**	559 ± 85	249 ± 80	192 ± 9	189 (125, 338)	349 (237, 612)
**Corn silage**	401 ± 32	392 ± 31	207 ± 15	352 (263, 535)	587 (439, 892)
**Tifton—85**	545 ± 37	216 ± 36	240 ± 16	357 (217, 934)	686 (428, 1782)
**Corn snaplage silage**	423 ± 52	375 ± 51	202 ± 8	277 (177, 567)	996 (561, 3455)
**Ryegrass hay**	533 ± 37	182 ± 36	284 ± 7	294 (164, 764)	515 (−7, 775)
**Alfalfa**	464 ± 22	102 ± 21	434 ± 11	335 (148, 2331)	741 (358, 5616)
**Pinto peanut**	494 ± 23	94 ± 21	411 ± 24	474 (166, 5542)	462 (300, 955)
***Zarzabacoa galana***	229 ± 42	165 ± 40	607 ± 9	260 (128, 1297)	579 (344, 1491)
**Florida hammock milkpea**	503 ± 15	55 ± 17	442 ± 11	259 (94, 2833)	1129 (465, 13584)
**Tropical kudzu**	381 ± 72	152 ± 62	467 ± 15	180 (91, 1582)	451 (233, 2248)
**Perennial horsegram**	444 ± 18	167 ± 25	388 ± 23	515 (283, 1789)	594 (156, 10051)
**Bluepea**	141 ± 19	338 ± 19	521 ± 13	324 (−7, 486)	397 (189, 3021)

1 $A_{1}^{\mathrm{std}}$ and $A_{2}^{\mathrm{std}}$ = standardized fast and slow potentially digestible fractions; $\Omega^{\mathrm{std}}$ = standardized long-term indigestible fraction. ${\tilde{\tau }}_{95\%}(\Omega )$ and ${\tilde{\tau }}_{99\%}(\Omega )$ = bootstrap median times (h) to reach 95% and 99% of asymptotic NDF degradation, respectively.

2 $A_{1}^{\mathrm{std}}$ , $A_{2}^{\mathrm{std}}$, and $\Omega^{\mathrm{std}}$ are on an aNDFom basis. Values in parentheses are 95% bias-corrected and accelerated (BCa) confidence intervals (3,000 bootstrap replicates).

### Times for completeness of degradation of digestible fiber mass

Median times to reach 95% $\left({\tilde{\tau }}_{95\%}(\Omega )\right)$ and 99% $\left({\tilde{\tau }}_{99\%}(\Omega )\right)$ of potential NDF degradation (pdNDF) varied substantially among specimens (Table 3). The shortest durations to 95% degradation were observed in Swaziland finger grass and Guinea grass, suggesting rapid overall digestion, whereas legumes like pinto peanut and horsegram required markedly longer periods. Median times to 99% degradation were consistently longer, particularly in species with high indigestible residue levels, such as alfalfa hay, Florida hammock milkpea, and perennial horsegram. Bias-corrected 95%BCa for these times were often wide, especially for those forages in which slow pool and indigestible fractions overrule, as in legumes and high-lignin grasses (Tables 1 and 3), underscoring estimation uncertainty under small sample sizes and highlighting the value of parametric simulation for robust inference.

### Prediction of the indigestible residue from lignin to NDF ratio

The top-supported model for predicting the iNDF residue (Ω) from the Lignin (sa):aNDFom ratio was the power-of-the-mean scaling law, fitted under normality (Table 4). The Gamma distribution model revealed significant study effects (*P* < 0.005) and study slopes (*P* < 0.005), requiring study-specific slopes (${\hat{\gamma }}_{11}=0.748\pm 0.1078$; ${\hat{\gamma }}_{12}=0.1228\pm 0.1678$; and ${\hat{\gamma }}_{13}=1.043\pm 0.0750$), and this heterogeneity makes a single overall exponent risky for broad inference. Confidence intervals revealed that ${\hat{\gamma }}_{11}$ and ${\hat{\gamma }}_{13}$ overlapped substantially (0.426–1.070 and 0.819–1.267, respectively), encompassing positive allometric, isometric, and negative allometric scaling, whereas ${\hat{\gamma }}_{12}$ was not different from zero (P=0.469), suggesting scale independence, possibly due to elevated uncertainty. Despite these limitations, practical prediction of the iNDF residue can be achieved using the fitted normal scaling equation (Figure 2). All alternative models received negligible support (Table 4). Diagnostic plots confirmed model adequacy: Pearson residuals adhered closely to theoretical normal quantiles in the Q-Q plot, with no evident outliers or systematic patterns (Figure 2a and b). The agreement plot (panel b) further demonstrated strong predictive performance, with a low discordance rate (0.05), most differences falling within the agreement limits, and only three discordant pairs.

**Figure 2:**
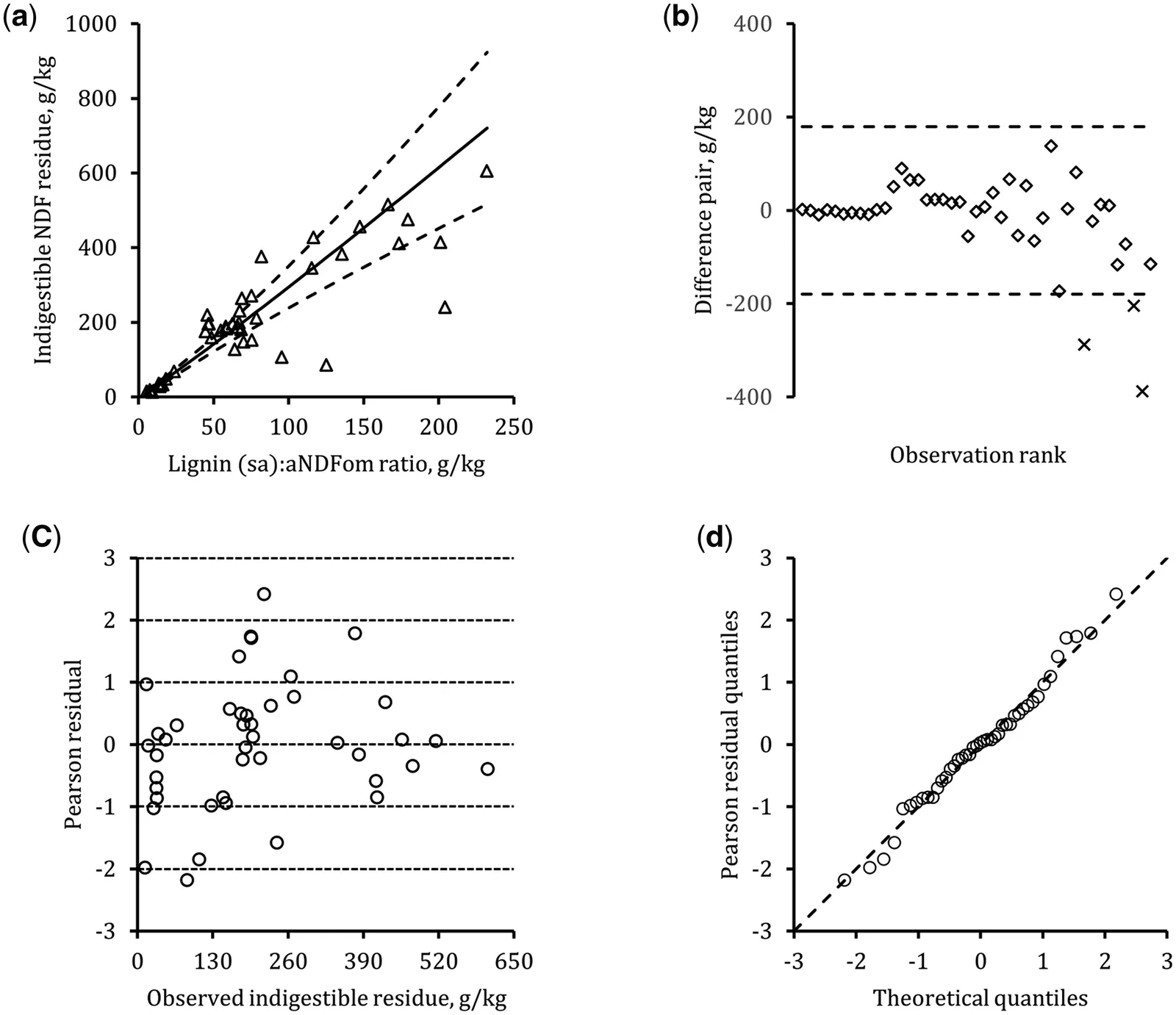
Scaling relationship between indigestible NDF residue $\left(\hat{\Omega },g/\text{kg}\right)$ estimated from long-term in vitro incubations and the Lignin (sa):NDF ratio. a) Shows observed values (open triangles), predicted values (solid line), and 99.5% confidence intervals (dashed lines); the inset highlights predictions for low ratio values. b) Presents concordant (open diamonds) and discordant (cross) difference pairs, and agreement limits (±180 g/kg, dashed lines) for a discordance rate of 0.05, and tolerance probability of 0.17 (n=43 observations). The x-axis shows the observation rank (ordered from lowest to highest observed indigestible NDF residue). c) Displays Pearson residuals (open circles) vs. observed values. d) Shows the normal Q-Q plot of Pearson residuals (ordinates) against theoretical quantiles (abscissas) with the dashed line (Y=X), indicating adherence to normality. Parameter estimates (mean ± SE) and 95% confidence intervals (CI) used for the fitted line in panel (a) are ${\hat{\gamma }}_{0}=2.22\pm 0.365\;g^{1-{\hat{\gamma }}_{1}}/{\text{kg}}^{1-{\hat{\gamma }}_{1}}$, CI: (1.13, 3.30); dimensionless ${\hat{\gamma }}_{1}=1.06\pm 0.045$, CI: (0.93, 1.19); $\hat{\sigma }=0.113\pm 0.0657\;g^{1-\hat{\psi }}/{\text{kg}}^{1-\hat{\psi }}$, CI: (-0.083, 0.308); and dimensionless $\hat{\psi }=1.193\pm 0.1135$, CI: (0.855, 1.530).

**Table 4:** Information criteria for the nonlinear scaling function fitted by maximum likelihood under normality (homoscedastic and heteroscedastic), lognormality, and gamma distributions, and using parameter estimates from Chandler et al. and Traxler et al. as inputs for Gauss quadrature approximation^1^.

Models	-l	${\mathrm{AICc}}_{r}$	$\Delta {\mathrm{AICc}}_{r}$	$w_{r}$	${\mathrm{ER}}_{r}$	$n_{\Theta_{r}}$
**Scaling function under normality, power-of-the-mean, minimized** -ℓ	224.1	457.3	0.0	0.918	1	4
**Gamma distribution, minimized** -ℓ	228.1	462.7	5.4	0.062	15	3
**Lognormal distribution, power-of-the-mean on log scale, minimized** -ℓ	228.1	465.3	8.0	0.017	55	4
**Lognormal distribution, minimized** -ℓ	231.0	468.5	11.2	0.003	270	3
**Chandler’s estimates as inputs under Lognormal distribution, no optimization**	235.1	476.8	19.5	$\approx 10^{-5}$	17154	3
**Scaling function under normality, minimized** -ℓ	246.8	500.2	42.9	$\approx 10^{-10}$	$\approx 10^{9}$	3
**Traxler’s estimates as inputs under Lognormal distribution, no optimization**	251.0	506.3	49.0	$\approx 10^{-11}$	$\approx 10^{10}$	3

1 -ℓ = negative logarithm of the likelihood function; ${\mathrm{AICc}}_{r}$ = Akaike information criterion estimated for the r-th model (r=1, 2,…, 7); $\Delta {\mathrm{AICc}}_{r}$ = Akaike differences between the r-th models; $w_{r}$ = Akaike weight or probability for the r-th model; ${ER}_{r}$ = evidence ratio of the r-th model; $n_{\Theta_{r}}$ = number of parameters for each r-th model, including variance parameters.

## Discussion

The chemical composition of forages provides critical insights into their nutritional quality, particularly through the balance between digestible and indigestible fractions, which influence intake, digestibility, and overall feed value for ruminants (Van Soest 1994; Mertens and Grant 2020). In this study, the multivariate analysis of major nutrients (DM, CP, CF, ash, aNDFom, Lignin (sa), and NFC) revealed distinct profiles among the 17 forage specimens, with grasses generally exhibiting greater aNDFom and lesser CP than legumes, consistent with known differences in fibrous carbohydrates and protein content (Van Soest 1994; Mertens and Grant 2020). The Lignin (sa):aNDFom ratio varied more than 5.5-fold (40 to 230 g/kg), highlighting the role of lignification in limiting fiber digestibility. Lignin-hemicellulose cross-links interact with cellulose in cell walls and reduce microbial access and enzymatic degradation (Jung et al. 1993; Wilson 1993, 1994; Wilson and Mertens 1995; Tedeschi et al. 2023; Dhakal et al. 2025). This ratio is an important predictor of indigestible residue (Chandler et al. 1980; Traxler et al. 1998; Van Soest et al. 2005, 2020), as demonstrated by the scaling relationship identified herein, but its accuracy depends on the precision and reproducibility of chemical and biological methods. Alternative NDF methods (e.g., non-pressurized immersion, or micro-NDF in sealed flasks) often lack specificity compared to reference AOAC procedures, leading to biased estimates of NDF due to method-matrix interactions, random effects, and heteroscedasticity (Silva et al. 2018; Hall and Mertens 2023). Moreover, the macromolecular structure of plant cell walls, comprising polysaccharides, proteins, phenolic compounds, and minerals, dictates their digestibility, with lignin incrustation in cell walls posing significant barriers to microbial degradation (Van Soest 1994; Dhakal et al. 2025; Tedeschi et al. 2025). Advancements in genetic manipulation of lignin pathways and processing methods (e.g., chemical, enzymatic, or microbial treatments) offer potential to enhance cell wall utilization, but require robust kinetic models and standardized in vitro techniques to accurately evaluate improvements in forage quality and animal performance (Lund et al. 2004; Mertens 2005; Hall and Mertens 2012; Foster et al. 2023; Dhakal et al. 2025; Tedeschi et al. 2025). The present results, consistent with previous observations, underscore the need for standardized reference chemical and biological methods in forage evaluation to ensure reliable relationships between chemical composition and fiber digestion kinetics.

Lignin (sa) is recognized as the most appropriate nutritional entity for predicting the iNDF. Unlike other fractions, Lignin (sa) behaves as a uniform nutritional entity, as validated by Lucas test evaluations, demonstrating a consistent relationship with the iNDF fraction across various forages. This uniformity stems from the fact that Lignin (sa) constitutes the incrusting and insoluble core of the cell wall, which acts as the primary physical barrier preventing microbial enzymatic access to structural polysaccharides (Krizsan and Huhtanen 2013; Van Soest et al. 2020).

In contrast, soluble or dispersible lignin (ΔL), defined as the arithmetic difference between Klason lignin (KL) and Lignin (sa), is characterized as a nutritionally heterogeneous fraction (Van Soest et al. 2020). Studies indicate that there is no significant correlation with fiber indigestibility, possessing a nutritional behavior distinct from the lignified core. While Lignin (sa) is strongly linked to the residue that limits digestion, it behaves as a component that can be released during ruminal fermentation without exerting an inhibitory effect on the final extent of NDF digestibility. Therefore, using KL as a quality predictor is less accurate, as it combines a functionally relevant fraction (Lignin (sa)) with a soluble fraction (ΔL) that does not dictate the fiber’s indigestibility value (Van Soest et al. 2020).

The nonideal nutritional heterogeneity of fiber, as conceptualized by Lucas (1964), necessitates kinetic modeling to dissect digestible and indigestible pools, rates of degradation, and passage dynamics, which collectively influence rumen fill, microbial growth, and overall digestibility (Van Soest et al. 2020; Tedeschi et al. 2023; Vieira et al. 2024). In this study, the top-supported G6G1G1 model effectively captured this heterogeneity. It incorporated time-dependent preparation (λ) and degradation rates ($\kappa_{1},\;\kappa_{2}$) for fast and slow pools, aligning with multicompartmental frameworks that account for variable residence times and selective retention of partially digested and indigestible residues in forage particles (Ahvenjärvi et al. 2001; Vieira et al. 2008a, 2008b, 2012, 2020; Tedeschi et al. 2012; Rohem Júnior et al. 2020). Such models expand upon Lucas’s static digestibility concept by simulating age-dependent processes, revealing problems in traditional models that, unfortunately, after several attempts, were ill-conditioned to our data and did not converge to feasible solutions.

The G6G1G1′s Gamma-distributed preparation times and fractional rates of degradation can be used to predict rumen fill and microbial N flow by integrating passage kinetics, as previously corroborated by marker transit and pool size data, reducing underestimation errors in microbial yield, fiber digestibility, pool sizes, and intake predictions (Vieira et al. 2008b, 2020, 2024). However, challenges persist, including methodological inconsistencies in fiber analysis (Silva et al. 2018; Hall and Mertens 2023), reproducibility in vitro, in situ, and perhaps pool and flow methods (Casali et al. 2008; Huhtanen et al. 2008b; Palmonari et al. 2016; Foster et al. 2023), and the need for new methods to enhance cell wall utilization (Tedeschi et al. 2023). Future models should incorporate these advances to refine predictions of ruminoreticular dynamics, ultimately providing quantitative traits to improve animal efficiency through selection (Ahvenjärvi et al. 2018).

The standardized parameters $A_{1}^{\mathrm{std}}$, $A_{2}^{\mathrm{std}}$, and $\Omega^{\mathrm{std}}$ provide a practical means to compute the potentially digestible (pdNDF) and indigestible fractions of aNDFom for each forage, free from experimental block effects. For example, in Coastal bermudagrass, the standardized fast-digesting pool ($A_{1}^{\mathrm{std}}=438\;$g/kg), slow-digesting pool ($A_{2}^{\mathrm{std}}=178\;$g/kg), and indigestible residue ($\Omega^{\mathrm{std}}=385\;$g/kg) sum to 1,000 g/kg (minor rounding error), yielding a potentially digestible fiber of 616 g/kg of aNDFom. These fractions can be used directly in mechanistic models to estimate rumen-available substrate and fill.

The parametric bootstrap estimates indicated that the median time required to reach 95% of potential NDF degradation (${\tilde{\tau }}_{95\%}$) was approximately 300 h across the forages examined. At this incubation time the residue consists of the entire asymptotic indigestible fraction (Ω, i.e., true iNDF) plus only a small residual portion of potentially digestible fiber. Thus, while ultra-long incubations remain the most direct way to approach the true asymptotic value under anaerobic conditions, an incubation of approximately 300 h already digests the great majority of the potentially digestible fiber, leaving a residue that constitutes a practical, high-quality operational estimate of undigested NDF (uNDF). This duration therefore represents a feasible compromise when the goal is to obtain a reliable estimate of the indigestible core for use in fill and digestibility predictions (Lippke et al. 1986; Casali et al. 2008; Krizsan and Huhtanen 2013; Mertens 2016; Palmonari et al. 2016). A well-estimated asymptotic iNDF remains valuable as a reference against which the closeness of any practical uNDF measurement can be judged.

A reliable estimate of the standardized asymptotic indigestible residue ($\Omega_{\mathrm{std}}$) is required for more than static characterization of feeds, digesta, or feces. Because the indigestible fraction occupies space in the ruminoreticulum and can disappear only by passage, it is a primary determinant of fill, effective digestibility under normal passage rates, and microbial protein supply (Mertens and Ely 1979; Mertens and Grant 2020). Dynamic simulations have shown that a 1% reduction in the indigestible fraction produces an approximately 1% increase in maximum digestible DM intake, an effect larger than that of a comparable change in digestion rate (Mertens and Ely 1979). Subsequent evaluations confirmed that the size of the potentially digestible pool is generally more important than digestion rate or lag for both digestibility and intake (Mertens and Ely 1982). As Mertens (2011) emphasized, the reason long fermentation times are used has nothing to do with the time feed spends in the rumen; these extended incubations are necessary to obtain an accurate estimate of the asymptotic indigestible residue so that the kinetics of the potentially digestible pools can be properly determined. When age-dependent digestion kinetics are integrated with passage, accurate values of $\Omega_{\mathrm{std}}$ improve predictions of ruminoreticular fiber mass, total-tract digestibility, and microbial protein flow (Vieira et al. 2008b, 2020, 2024). The long-term kinetic estimation of $\Omega_{\mathrm{std}}$ therefore supplies the reference value required for realistic dynamic predictions and the benchmark against which practical operational estimates, such as uNDF obtained near the median ${\tilde{\tau }}_{95\%}$ of approximately 300 h, can be evaluated (Lippke et al. 1986; Casali et al. 2008; Krizsan and Huhtanen 2013; Mertens 2016; Palmonari et al. 2016).

Despite the robust estimation of indigestible residues (iNDF) from long-term in vitro kinetics and the identification of a scaling relationship with lignin content, several limitations must be acknowledged. First, the study was based on a set of 17 forage specimens. Although this number is modest, it is comparable to the data sets that underpinned the original 2.4 × lignin factor widely used in nutrition models (Chandler et al. 1980) and its subsequent evaluations (Traxler et al. 1998; Van Soest et al. 2005). Nevertheless, the limited number of samples, particularly for C3 grasses, still restricts the generality of the findings and of the scaling function across a wider range of forages, maturities, and growing conditions (Casali et al. 2008; Palmonari et al. 2016; Foster et al. 2023). Second, the kinetic parameters and the indigestible residues reported in the present study were obtained under long-term in vitro batch conditions. Although the scaling law incorporated indigestible residue estimates previously obtained in situ (Vieira et al. 2012), no independent in vivo or in situ empirical evaluation of either the kinetic parameters or the scaling function was performed. It is important to emphasize that the indigestible core of fiber cannot be measured directly in the animal. The indigestible fraction is an operationally defined entity that can only be estimated by exhaustive anaerobic incubation of feed, digesta (extrusa, ruminal, omasal, abomasal or duodenal), or fecal samples under in vitro, continuous culture, or in situ (in sacco) conditions (Chandler et al. 1980; Huhtanen et al. 2008a; Allen 2019; Foster et al. 2023). Third, even after careful removal of 20.1% of the original observations that exhibited clear non-biological increases in residual mass (methodological artifacts, mostly at incubation times greater than 500 h) in the present study, residual uncertainty persists in the slow-digesting pools and in the very long incubation times required to approach the asymptote. This uncertainty is reflected in the wide parametric bootstrap confidence intervals (95% BCa) for the median times needed to reach 95% and 99% of potential NDF degradation, particularly in high-lignin legumes. Fourth, the NDF residues used for the kinetic analyses were deliberately determined without sodium sulfite to allow direct comparison with historical long-term incubation data (Chandler et al. 1980; Vieira et al. 2012); this choice, while justified for comparability, may have reduced analytical precision relative to the standardized aNDFom procedure (Hall and Mertens 2012). Finally, the scaling law, although statistically well supported within the present data set, still requires independent empirical evaluation across a broader range of forage species, maturities, and environmental conditions before it can be recommended for routine use in nutrition models (Silva et al. 2018; Hall and Mertens 2023).

Regardless of the weakness arising from both the accuracy and precision natures, the scaling function identified in this study offers several practical advantages for predicting indigestible aNDFom (iNDF), particularly in mechanistic nutrition models and in research focused on ruminal pool sizes and nutrient flows (Ahvenjärvi et al. 2001; Huhtanen et al. 2006, 2008a; Mertens 2016, Tedeschi and Fox 2020; Vieira et al. 2024). By leveraging the Lignin (sa)/aNDFom ratio, which is a readily obtainable chemical measure, the function enables rapid, non-destructive estimation of indigestible fractions when resource-intensive long-term in vitro or in situ incubations are not at hand and are prone to methodological biases (Hall and Mertens 2012, 2023; Silva et al. 2018). This predictive capability integrates adequately with mechanistic frameworks, allowing accurate parameterization of rumen submodels to forecast microbial protein synthesis, VFA production, and energy yield from fiber fermentation, as the indigestible residue directly influences substrate availability and digestion kinetics (Mertens 2016; Mertens and Grant 2020; Tedeschi and Fox 2020; Vieira et al. 2024). If this equation or estimation tactics became valid for pool and flow methods, it might represent a faster way to predict pools, perhaps reducing errors from heterogeneous fiber behavior and improving simulations of selective retention, intake regulation, and total tract digestibility (Huhtanen et al. 2008a, 2014; Allen 2023; Tedeschi et al. 2023; Vieira et al. 2024).

This study advances the kinetic evaluation of forage fiber by demonstrating that the generalized compartmental model GηG1G1 provided a superior description of heterogeneous biphasic degradation profiles in a broad range of tropical and temperate forages. The derivation and statistical evaluation of the scaling law ${\hat{\Omega }}^{\mathrm{std}}={\hat{\gamma }}_{0}{\times (\text{Lignin}\;(\text{sa})/\text{aNDFom})}^{{\hat{\gamma }}_{1}}$ (${\hat{\gamma }}_{0}=2.22\pm 0.365\;g^{1-{\hat{\gamma }}_{1}}/{\text{kg}}^{1-{\hat{\gamma }}_{1}}$ with a CI: (1.13, 3.30), and ${\hat{\gamma }}_{1}=1.06\pm 0.045$ with a 95% CI: 0.93–1.19), further offers a practical and robust alternative to long-term incubations (up to 2,500 h) that fully exhaust slow-digesting pools, supporting a resilient isometric relationship between the indigestible core and lignification and extending the linear assumption originally proposed by Van Soest (Chandler et al. 1980; Van Soest et al. 2005).

## Conclusions

This study provides a comprehensive mechanistic framework for estimating the indigestible core of forage fiber using long-term in vitro kinetics and a predictive scaling function based on the Lignin (sa):aNDFom ratio, accounting for fiber heterogeneity through robust statistical inference. The generalized compartmental model GηG1G1, with η=6 specifically selected via I-T criteria, effectively captures biphasic degradation patterns, dynamic lags, and asymptotic residues across 17 diverse forage specimens. Parametric bootstrapping indicates that approximately 300 h are sufficient to exhaust 95% of potentially digestible fiber in vitro, suggesting practical incubation durations to estimate the indigestible core of fiber despite uncertainty in slow pools. In addition, despite the large variability in the prediction of the indigestible core at greater Lignin (sa):aNDFom ratios, the isometric relationship between the asymptotic indigestible residue of fiber and lignification endures. Limitations, such as the modest number of forage specimens and residual uncertainty associated with long-term in vitro estimates, highlight the need for broader independent empirical evaluation and greater efforts toward standardization of chemical and biological procedures.

## Abbreviations:

$A^{\mathrm{std}}$: standardized single fraction of potentially digestible fiber
$A_{1}^{\mathrm{std}}$: standardized fast potentially digestible fraction of fiber
$A_{2}^{\mathrm{std}}$: standardized slow potentially digestible fraction of fiber
${\mathrm{AICc}}_{r}$: Akaike information criterion corrected for small samples
${\Delta \mathrm{AICc}}_{r}$: Akaike difference
aNDFom: amylase-treated, ash-corrected neutral detergent fiber
BCa 95%: bias-corrected 95% confidence intervals
CAR: continuous autoregressive correlation structure
CF: crude fat
CP: crude protein
DM: dry matter
${ER}_{r}$: evidence ratio of the r-th model
EXP 1: homogeneous exponential degrading model
EXP 2: two-pool exponential degrading model
G6G1G1: two-pool compartmental model with 6th-order time-dependency
GηG1: single-pool Gamma time-dependency model
GηG1G1: two-pool Gamma time-dependency model
GMM1: single-pool generalized Michaelis-Menten model
GMM2: two-pool generalized Michaelis-Menten model
I-T: Information-theoretic approach
iNDF: asymptotic indigestible neutral detergent fiber (corresponding to the model parameter Ω )
KL: Klason lignin
Lignin (sa): lignin determined by solubilization of cellulose with 72% sulfuric acid
-ℓ: negative logarithm of the likelihood function
MANOVA: multivariate analysis of variance
N: Nitrogen
NDF: ash-corrected neutral detergent fiber of the in vitro incubation residue determined without Na₂SO₃
NFC: non-fibrous carbohydrates
$n_{\Theta_{r}}$: number of parameters for each r-th model, including variance parameters
pdNDF: potentially digestible NDF
POM: power-of-the-mean variance function
%RSD: relative standard deviation
SEM: standard error of the mean
uNDF: undigested neutral detergent fiber remaining after a defined incubation time
VFA: volatile fatty acids
$w_{r}$: model probabilities or Akaike weights
$\gamma_{1}$: dimensionless power parameter for the scaling law
$\kappa_{i}$: fractional degradation rates of the i-pool
η: dimensionless order of time-dependency
λ: rate parameter of substrate preparation
τ: incubation time
Ω: asymptotic indigestible NDF estimated by the kinetic models (equivalent to iNDF)
$\Omega^{\mathrm{std}}$: standardized indigestible fraction of fiber
